# Exposure to indoor air pollution and the cognitive functioning of elderly rural women: a cross-sectional study using LASI data, India

**DOI:** 10.1186/s12889-022-14749-7

**Published:** 2022-12-05

**Authors:** Manoj Dakua, Ranjan Karmakar, Papai Barman

**Affiliations:** International Institute for Population Sciences, Deonar, Mumbai, 400088 India

**Keywords:** Indoor air pollution, Cognitive functional health, Older adults, LASI

## Abstract

**Background:**

The majority of people in rural developing counties still rely on unclean and solid fuels for cooking, putting their health at risk. Adult and elderly women are most vulnerable due to prolonged exposure in cooking areas, and Indoor Air Pollution (IAP) may negatively impact their health and cognitive function. This study examines the effect of IAP on the cognitive function of middle-aged and elderly rural women in India.

**Methods:**

The study utilized the data from the Longitudinal Ageing Study in India (LASI 2017–18, Wave-1). Bivariate analysis and multilevel linear regression models were applied to show the association between IAP and the cognitive abilities of rural women and results from regression were presented by beta coefficient (β) with 95% confidence interval (CI). Confounding factors such as age, education, health risk behaviours, marital status, monthly per capita consumption expenditure (MPCE), religion etc. were adjusted in the final model.

**Results:**

The study found that 18.71 percent of the rural women (*n* = 3,740) lived in Indoor Air Pollution exposed households. IAP was significantly found to be associated with the cognitive functional abilities among the middle and older aged rural women. Middle and older aged rural women exposed to IAP had lower cognitive functional abilities than non-exposed women. Comparing to the non-exposed group, the cognitive score was worse for those exposed to IAP in both the unadjusted (β = -1.96; 95%CI: -2.22 to -1.71) and the adjusted (β = -0.72; 95%CI: -0.92 to -0.51) models. Elderly rural women from lower socioeconomic backgrounds were more likely to have cognitive impairment as a result of IAP.

**Conclusion:**

Findings revealed that IAP from solid fuels could significantly affect the cognitive health of elderly rural women in India, indicating the need for immediate intervention efforts to reduce the use of solid fuels, IAP and associated health problems.

**Supplementary Information:**

The online version contains supplementary material available at 10.1186/s12889-022-14749-7.

## Background

Air pollution is the fourth leading cause of death worldwide in 2019, with 6.67 million deaths from it and 3.6 percent of the disability-adjusted life-years (DALYs) risk factors from Indoor Air Pollution (IAP), also known as household pollution [1]. Air pollution mostly generated by human activities has a detrimental effect on physical and mental health of human [2, 3]. Indoor Air Pollution is a significant environmental risk factor for a variety of respiratory illnesses, including acute and chronic respiratory infections, cancer [4–6] cardiovascular diseases, low birth weight, stillbirth, Tuberculosis (TB) and asthma, cataracts, and blindness [7–9]. World Health Organization (2019) reported that nearly 90 percent of people worldwide breathe polluted air**.** IAP is frequently regarded as one of the most significant factors of illness and mortality worldwide [1]. And it is worse in developing countries because people use dirty fuels like wood, animal dung, or crop wastes for cooking at home, especially in rural regions [10, 11]. Furthermore, burning biomass produces smoke which is one of the environmental issues mostly prevalent in developing countries [12]. Indoor air pollution is responsible for four percent of the worldwide burden of disease, primarily due to unclean cooking fuels which adversely effects on health of women, and elderly people spending a long time in cooking areas [11, 13, 14]. For domestic cooking, unclean biomass fuels are widely used, especially in developing countries and India. Along with the adverse effect on respiratory illnesses, cardiovascular diseases, low birth weight, stillbirth, cataracts, and blindness, it also effects on depression and cognitive dysfunction among adult and elderly people, especially on women [10, 15, 16].

Cognitive function is mental capabilities which is essential for day-to-day life activities; it contains memory, orientation, verbal fluency, arithmetic abilities, executive function, and object identification. It is associated with the health, disabilities, and well-being of older people [10, 15]. Previous studies have exhibited the positive association between cognitive functioning abilities, health, and wellbeing [17, 18]. Meanwhile, cognitive dysfunction is recognized as the risk factor, leading to disabilities and even death [19–21]. Previous research has also manifested an adverse association between cognitive functioning ability and age [22, 23]. With the increasing life expectancy and number of elderly populations after 60 and above age, low cognitive functioning ability, especially dementia has been found to become epidemic, as revealed by the world health organization’s recent report [24]. According to the Indian Census reports, the elderly population grew from 5.6 percent in 1961 to 8.6 percent in 2011 and is expected to rise to 19.5 percent by 2050 [25]. Nearly 20 in 1000 elderly population is found to be suffered from dementia in India [26]. Moreover, the prevalence of dementia is reported higher among the female elderly population [27]. The demand for intensive and long-term care for dysfunctional older populations grows as the proportions of the elderly population increases, putting a strain on India’s healthcare system [28]. These circumstances underline the critical need for cognitive dysfunction screening, which will eventually help healthcare providers detect people who are at risk of dementia, especially among the women elderly population having higher life expectancy, percentage share in total elderly population, and long term-exposure to indoor air pollution. In Indian patriarchal culture women spend a prolonged time for doing household chores work and preparing meal for other members, while men are responsible for earning. Therefore, women are more prone to expose to indoor air pollution than men. A study also mentioned that in India, women particularly from rural area are more subjected to their prolonged exposure to indoor air pollution with the task of cooking with unclean or solid fuels for domestic purposes [10, 15, 29]. According to the National Family Health Survey 2019–21, a large share (40.6 percent) of households from rural area do not have access to clean fuel for domestic cooking, which exposes them to a higher risk of physical and cognitive disability [30].

A substantial previous works focused on the gender dimension and cognitive functioning health among the elderly population, looking at the individual and socio-economic factors [18, 22, 31, 32]. Hardly did previous studies focus on the environment factor where a woman spends her whole life after marriage and link between ambient air pollution and individual cognitive ability [33, 34]. In India, considering the increasing number of women and the prevalence of dementia among the 60 and above population and the higher exposure to indoor air pollution, we anticipated that female elderly population are the most vulnerable group of poor cognitive functioning health. Therefore, reducing the health problem and increasing the quality of life of the women residing in the rural areas and subjected to a higher risk, the current study aimed to investigate the association between indoor air pollution and cognitive abilities in India.

## Methods

### Study design and study population

The data for this study came from Wave 1 of the Longitudinal Ageing Study of India (LASI), which was gathered in 2017–18. LASI is the world's most extensive longitudinal survey of middle- and older persons, as well as India’s first. The LASI survey was developed in collaboration with the International Institute for Population Sciences (IIPS), Harvard T. H. Chan School of Public Health (HSPH), and the University of Southern California (USC). It was designed to provide scientific data on the health, economic, and social determinants of the country's older individuals [35]. LASI will be performed every two years for the next 25 years. Wave 1 of the LASI survey covered 72,250 individual samples aged 45 and above and spouses from the whole country except Sikkim state. The survey applied the multistage stratified area probability cluster sampling with response rates ranging from 96 percent in Nagaland to 74 percent in Chandigarh. In rural regions, three-stage sampling was employed, while in urban areas four-stage sampling was used. The LASI 2020 final report goes over the sampling design, data collection, survey execution, and validation, and for further detail [35].

The survey identified 44,462 age-eligible households and 82,650 age-eligible individuals and interviewed 42,949 households and 72,250 individuals. For this study, we have chosen rural as the respondent's location and female as the respondent's gender. The study’s final adequate sample size was 22,535 individuals aged 45 or above.

### Outcome variable

The LASI survey used the following five domains to assess the cognitive functional health of middle and older aged adults:

- Memory: Immediate word recall (score 0–10) and delayed word recall (score 0–10) were used to assess memory. The total memory score ranged from 0 to 20.
- Orientation: The orientation was measured using the time (score 0–4) and place (score 0–4). The overall score for orientation ranged from 0 to 8.
- Arithmetic function: Backward counting (score 0–2), serial 7 (score 0–5), and computation were used to evaluate the arithmetic function (score 0–2). The arithmetic function's overall score ranged from 0 to 9.
- Executive function: Paper folding (scoring 0–3) and pentagon drawing (score 0–1) was used to assess executive function. The overall score for executive function ranged from 0 to 4.
- Object naming: The scores for object naming ranged from 0 to 2.

A composite cognitive index was constructed by combining memory orientation, arithmetic function, executive function, and object naming scores. A simple count approach was used to calculate the index, where the selected items were summed. The overall score of the composite cognitive index varied from 0 to 43 exhibited higher compositive cognitive score, better cognitive ability and vice versa. See the LASI 2020 report for further information on the cognitive function assessment [35].

### Explanatory variable

Household exposed to indoor air pollution by kitchen configurations among rural middle and olderly women was the key explanatory variable. Physical, chemical, and biological contamination of interior air are included under Indoor Air Pollution. During the LASI survey (2020), one separate section was canvassed on Indoor Air Pollution. The explanatory variable i.e., IAP was measured by combining 5 questions canvassed in the LASI survey. Two questions regarding the fuel used for cooking and other purpose were asked, i.e., (1). What is your main source of cooking fuel? and (2) What are those other sources of fuel used for other purposes (such as boiling water for bathing, lighting, etc.)? (Responses: Liquefied Petroleum Gas (LPG)-1, Biogas-2, Kerosene-3, Electric-4, Charcoal/Lignite/Coal-5, Crop residue-6, Wood/Shrub-7, Dung cake-8, Do not cook at home-9, Other, please specify-10) We recoded both the variables and considered LPG, Biogas, and Electric as clean fuels and all others as unclean or solid fuels. The next question was on the type of oven used i.e., (3) In this household, is food MOSTLY cooked on a mechanical stove, on a traditional *Chullah* or over an open fire? (Responses: Mechanical Stove/Improved cook stove-1, Traditional chullah-2, Open fire-3, Other, please specify-4). We considered traditional *Chullah* and opened fire as the most pollution-generating source. Two questions pertaining to place of cooking and ventilation were asked during the survey, which follows- (4) Is the cooking usually done in the house, in a separate building, or outdoors? (Responses: In the house-1, In a separate building-2, Outdoors-3, Other, please specify-4); (5) Is the cooking mainly done under a traditional chimney, exhaust fan, electric chimney or near window/door? (Responses: Traditional chimney-1, Electric chimney-2, Exhaust fan-3, Near window/door-4, None-5). We considered in-house cooking with no ventilation system as vulnerable. Finally, all five factors were combined after recoding all five variables to differentiate IAP exposed and non-exposed households. We considered IAP exposed households as those who were using unclean sources for cooking and other than cooking by using traditional *chullah* or open fire and cooking inhouse without any ventilation system. Thus, households that use unclean and solid fuels such as crop residue, wood/shrub, dung cake, kerosene, charcoal/lignite/coal, etc., for domestic activities such as cooking, water boiling, room heating, bathing, etc., inside the house, cooking in traditional stoves or open space without a ventilation system is defined as having Indoor Air Pollution [10, 35]. The LASI national Report [35] provides more thorough information on Indoor Air Pollution caused by unclean or solid cooking fuels.

### Covariates

The analysis considers potential covariates of cognitive function at the individual and household levels, such as economic factors, health risk behaviour, and demographic factors. The age was classified into two groups: 45–59 middle-aged and 60 and above older adults. Marital status was categorized into two categories: currently with partner/husband and currently single. Currently married and live-in relationships are included in the currently with partner/husband category, and the currently single category includes widowed, divorced, separated, deserted, and never-married women. Years of schooling were recoded as 0 to indicate illiteracy, 1 represents primary school (1–5 years), 2 represents secondary school (6–9 years), and 3 represents higher secondary and above (10 years or above). Living status was divided into two categories: single and not single. Self-reported smoking, smokeless tobacco use, and alcohol consumption were categorised into two categories: ever had and never had. The respondents' self-reported health status was divided into good, fair, and poor. Sleeping problems include both staying asleep or sleeping in a non-restorative manner. This study defined the sleeping problem as having symptom-based sleep disruptions [35]. India is a vastly diverse country, and religion and geographical differences significantly impact people's health. As per previous studies, certain religions have a poor health status [36, 37]. Therefore, this study included religion and geographic regions (defined by the census regional division) as covariates.

### Analytical approach

The study applied the multilevel linear regression model to investigate the link between Indoor Air Pollution and middle and older-aged women's cognitive functioning abilities. On the first level, individual-level effects were shown, while household levels were shown on the second. Four sub-sequential step approaches were used in the investigation. First, a null model was fitted (model- I). Individual and household-level characteristics were then adjusted separately in model II and model III. Individual level characteristics such as age, marital status, years of schooling, living status, health risk behaviours, self-reported health, and sleeping problems were considered to be adjusted for confounders in model II. Household level characteristics such as mean month consumption expenditure, religion, and regions were considered in model III. The final model was fitted after adjusting the individual and household level characteristics in the model-IV. Cognitive mean scores under each domine and composite score were presented with a 95% confidence interval among the exposure and non-exposure groups. All variables were evaluated for multicollinearity before being included in the regression models. The regression coefficients were presented with a 95 percent confidence interval. All the statistical computations were done by STATA 14.1.

## Results

### Background characteristics of the rural Middle-aged and older women

Since all the works were carried out using weight, only weighted values were interpreted. Table 1 presents the study samples’ demographic, socio-economic, general health, and exposure to Indoor Air Pollution characteristics to understand distribution. Half of the respondents were elderly aged 60 and above. Out of the total of 22,535 respondents, a majority, around 63 percent lived with their partner or spouse. Nearly half of the older women (43%) came from poor MPCE families. While, a significant share of the older women (83.82%) belonged to the Hindu religion, Muslim only accounted for 10 percent and Christian and other religions comprised around 2 and 3 percent respectively. In terms of years of schooling, most of the respondents (77%) had no formal education, while only 13 percent completed primary schooling. A substantial respondent, nearly 94 percent lived with someone. Regarding health behaviour, more than one-fifth said they smoked or used smokeless tobacco, whereas only 3 percent declared that they took alcohol. Similarly, a considerable percentage (96%) reported that they had no sleeping problem. Nearly one-tenth of the respondent reported that their general health was poor. Considering Indoor Air Pollution, nearly 19 percent of the respondents were living in such households.

**Table 1:** Background characteristics of the rural middle-aged and older adults’ aged rural women in India, LASI, 2017–18

Characteristics	unweighted percentage	Weighted percentage	Total number of samples (*n* = 22,535)
***Age***			
*45–59*	52.75	50.01	11,887
≥ *60*	47.25	49.99	10,648
***Marital status***			
*Currently with partner/husband*	64.30	63.53	14,489
*Currently single*	35.70	36.47	8045
***MPCE***			
*Poorer*	39.88	43.08	8988
*Non-poorer*	60.12	56.92	13,547
***Religion***			
*Hindu*	74.60	83.82	16,603
*Muslim*	9.65	9.64	2147
*Christian*	10.82	2.97	2408
*Other*	4.93	3.58	1097
***Region***			
*North*	17.79	12.79	4010
*Central*	16.49	20.83	3715
*East*	19.07	24.81	4298
*North East*	10.51	1.21	2368
*West*	13.13	15.38	2959
*South*	23.01	24.98	5185
***Years of schooling***			
*0*	72.93	77.12	16,434
*1–5*	14.33	13.15	3229
*6–9*	7.83	6.49	1764
*10* +	4.92	3.24	1108
***Living status***			
*Alone*	5.49	6.26	1237
*Not alone*	94.51	93.74	21,298
***Ever smoked or smokeless tobacco***			
*Yes*	23.47	22.98	5259
*No*	76.53	77.02	17,148
***Ever consumed alcohol***			
*Yes*	5.50	3.39	1233
*No*	94.5	96.61	21,183
***Sleeping problems***			
*Present*	3.88	4.27	873
*Absent*	96.12	95.73	21,610
***Self-rated health***			
*Good*	57.41	54.40	12,762
*Faire*	30.78	32.84	6842
*Bad*	11.82	12.76	2627
***IAP***			
*Exposed*	16.85	18.67	3740
*Non-exposed*	83.15	81.33	18,462

Note: number of observations are not equal because of missing cases

### Descriptive results

Table 2 shows the cognitive functioning health of exposed and non-exposed women in rural settings. It shows unweighted mean values exhibiting higher average values and better cognitive functioning health. A cconfidence interval was employed to see the significant level of the difference, and all the values were found to be statistically significant indicating that differences among the comparison groups were significant. The mean values were observed lower among the exposed women than non-exposed women in each domain. Based on the memory test, the mean value was 8.55 among the exposed women, while 9.29 among the non-exposed women. Similarly, the mean values of orientation, arithmetic and executive functioning, and object naming test were 5.87, 2.46, 1.90, and 1.86, respectively; among the IAR exposed women and the values were lower than non-exposed women. The overall mean composite value was also found to be lower (mean: 20.64) among the exposed women than its counterpart (mean: 22.61).

**Table 2:** Domain wise cognitive ability scores among rural middle-aged and older adults’ women in India, LASI, 2017–18

	**Non-exposed group** **(*n* = 3740)**		**Exposed group** **(*n* = 18,454)**	
**Domain**	Mean	95% Confidence interval	Mean	95% Confidence interval
Memory (0–20)	9.29	9.23–9.34	8.55	8.43–8.67
Orientation (0–8)	6.17	6.15–6.19	5.87	5.81–5.92
Arithmetic function (0–9)	3.15	3.11–3.19	2.46	2.39–2.54
Executive function (0–4)	2.10	2.09–2.12	1.90	1.86–1.93
Object naming (0–2)	1.89	1.88–1.89	1.86	1.84–1.88
Composite cognitive index (0–43)	22.61	22.51–22.72	20.64	20.42–20.87

**Fig. 1:**
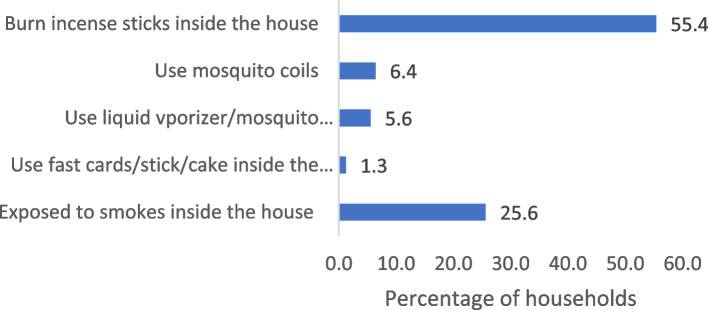
Percentage of households utilise various products in their house daily, India, LASI, 2017–18

Figure 1 depicts the percentage of households utilising various products in their homes daily. These events increase exposure to indoor air pollution. A majority of the rural women (55.41%) were engaged in burning incense sticks inside their houses. Almost a quarter of the respondents were exposed to smoke inside the house. Practicing mosquito coils and liquid vaporizer/mosquito repellent/mats were found 6 and 5 percent among the rural women respectively. Very few respondents (1.25%) reported using fast cards/stick/cake inside the house.

**Fig. 2:**
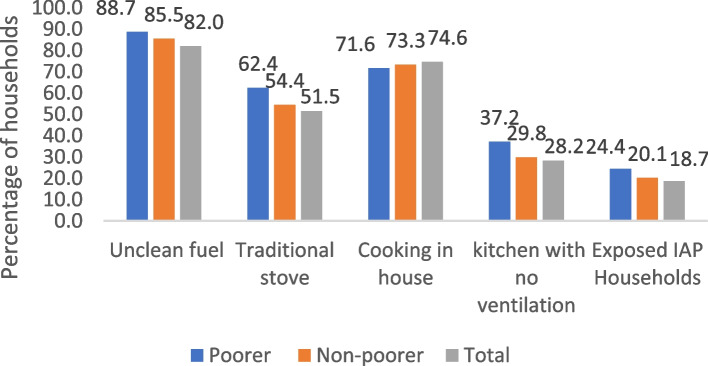
Percentage of households utilising various components of indoor air pollution, India, LASI, 2017–18

Figure 2 depicts the percentage of households utilising various elements related to indoor air pollution. It was estimated that nearly 82 percent of households practiced unclean fuel, whereas poorer were more exposed than non-poorer (88.68% vs. 85.48%). Utilization of traditional stoves was higher (62.42% vs. 54.43%) among the rural poorer than the non-poorer women. Cooking within household is universal irrespective of poorer and non-poorer groups and nearly 74 percent of rural women were found to cook within household. Around 37 percent of poorer households had no ventilation system in the kitchen, while the value was around 30 percent for non-poorer households. Overall, nearly 18.67 percent of rural households were exposed to indoor air pollution, whereas around 25 percent of poorer and 20.09 percent of non-poorer households were exposed to indoor air pollution.

### Association of indoor air pollution and cognitive abilities among rural middle-aged and older adult women

Adjusted and unadjusted associations between indoor air pollution and cognitive functioning health are shown in Table 3. β coefficient and confidence interval at 95 percent level were estimated to understand the association and its significant level respectively. Four separate models were employed: model I for unadjusted, model II for adjusted by individual characteristics, model III for adjusted by household characteristics, and model IV for adjusted by individual and household characteristics. In the model I, β coefficient was negative, indicating that exposed women were less likely (β = -1.96; 95% CI = -2.22, -1.71) to have good cognitive functioning health, which was statistically significant at < 0.001. In model II, after adjusting the individual characteristics, the β coefficient was also found to be significantly negative (β = -0.76; 95% CI = 0.96, -0.56), although the value is seen as lower than the unadjusted one. A similar pattern was observed in model III where the β coefficient was significantly negative (β = -1.69; 95% CI = -1.95, -1.43), which was higher than model II and slightly lower than the model I. The same pattern was observed while dealing with model IV. Overall, one may say that whether adjusted or unadjusted, the rural women exposed to indoor air pollution were more likely to have poor cognitive functioning health compared to non-exposed women.

**Table 3:** Multilevel analysis of composite cognitive ability score and indoor air exposure with adjusting the individual and household level factors among rural elderly women, LASI, 2017–18

Characteristics	Model-I unadjusted beta coefficient	Model-II adjusted beta coefficient (individual characteristics)	Model-III adjusted beta coefficient (household characteristics)	Model-IV adjusted beta coefficient (individual and household characteristics)
	β (95%CI)	β (95%CI)	β (95%CI)	β (95%CI)
**Indoor Air pollution**				
Exposure	-1.96 ***(-2.22 to -1.71)	-0.76*** (-0.96 to -0.56)	-1.69*** (-1.95 to -1.43)	-0.72*** (-0.92 to -0.51)
Not exposure^a^				
**Age**				
45–59^a^				
≥ 60		-2.11*** (-2.27 to -1.94)		-2.09*** (-2.25 to -1.93)
**Marital status**				
Currently with partner/husband^a^				
Currently single		-1.28*** (-1.45 to -1.11)		-1.26*** (-1.43 to -1.09)
**Years of schooling**				
0^a^				
1–5		4.48*** (4.26 to 4.69)		4.40*** (4.18 to 4.62)
6–9		7.82 ***(7.53 to 8.10)		7.64*** (7.35 to 7.92)
10 +		10.54*** (10.19 to 10.89)		10.25*** (9.89 to 10.61)
**Ever smoked or smokeless tobacco**				
Yes		-0.40*** (-0.58 to -0.22)		-0.28** (-0.46 to -0,10)
No^a^				
**Ever consumed alcohol**				
Yes		-1.95*** (-2.28 to -1.61)		-1.84*** (-2.17 to -1.50)
No^a^				
**Living status**				
Alone		0.26 (-0.08 to -0.60)		0.09 (-0.24 to 0.43)
Not alone^a^				
**Sleeping problems**				
Present		-.68*** (-1.07 to -0.28)		-0.65*** (-1.04 to -0.26)
Absent^a^				
**Self-rated health**				
Good		1.69***(1.45 to 1.94)		1.86*** (1.61 to 2.10)
Faire		1.18*** (0.92 to 1.44)		1.23*** (0.98 to 1.49)
Bad^a^				
**MPCE**				
Poorer			-2.04*** (-2.24 to -1.84)	-0.76*** (-0.92 to -0.60)
Non-poorer^a^				
**Religion**				
Hindu			-0.63** (-1.09 to -1.68)	-0.34 (-0.71 to 0.02)
Muslim			-1.75*** (-2.29 to -1.20)	-0.82*** (-1.25 to -0.40)
Christian			-0.75** (-1.31 to -0.19)	-0.96*** (-1.39 to -0.52)
Other^a,b^				
**Region**				
North			-1.02*** (-1.34 to -0.72)	-0.47***(-0.72 to -0.23)
Central			-1.52*** (-1.84 to -1.21)	-0.85*** (-1.10 to -0,61)
East			-0.12 (-0.43 to 0.17)	0.22 (-0.01 to 0.46)
North East			-0.12*** (-1.26 to -0.45)	-0.63*** (-0.96 to -0.31)
West			-1.53*** (-1.26 to -1.20)	-0.68*** (-0.95 to -0.42)
South^@^				

^a^Reference categories; ***p < 0.001, **p < 0.01, *p < 0.05; ^b^Sikh, Buddhist/Neo-Buddhist, Jain, Jewish, Parsi/Zoroastrian, No religion, Any other religion

Looking at the individual characteristics, the behavioural health risk factors played a significant role in improving the cognitive mean functional score among middle and older rural women. Compared to the participants who never consumed alcohol, ever consumed respondents (β = -1.95; 95% CI = -2.28, -1.68) had significantly (*p* =  < 0.001) lower mean cognitive functioning score. Participants who ever took smoked or smokeless tobacco also had significantly lower mean cognitive score (β = -0.40; 95% CI = -0.58, -0.22) than respondents who never smoked or smokeless tobacco. Compared to middle-aged women (45–59 years), older women (60 and above years) had significantly lower mean cognitive functional score (β = -2.11; 95% CI = -2.27, -1.94), implying that rural women were more prone to be suffered from poor cognitive functioning health by IAP. Years of schooling had a significant positive role on cognitive function implying that with increasing years of schooling significantly mean cognitive mean score increased. Participant living as single had less mean cognitive score (β = -1.28; 95% CI = -1.45, -1.11) than their counterparts. The mean score of cognitive function (β = 1.69; 95% CI = 1.45, 1.94) was higher with self-rated good health than those in self-rated poorer health.

The mean score of cognitive functioning health was lower (β = -2.04; 95% CI = -2.24, -1.84) among participants who belonged to the poor category than their counterparts. Across the geographic regions and religions, the mean cogitative functioning scores were found to be varied. Compared to the southern region, the mean cognitive scores were significantly lower in all others regions. Compared to the participants belonged to the other religion, Muslim (β = -1.75; 95% CI = -2.29, -1.20), Hindu (β = -0.63; 95% CI = -1.09, -1.68), and Christian (β = -0.75; 95% CI = -1.31, -0.19) had significantly lower mean cognitive scores.

## Discussion

### Summary and findings

Access to clean cooking fuel and technologies has remained health, gender, economic, environmental, and climate issue for the last three decades. Almost three billion people lack modern cooking facilities and are primarily dependent on unclean or polluting energy sources for daily household chores [38]. The Sustainable Development Goal on energy (SDG 7) aims to ensure universal access to clean fuel and technologies. Achieving this objective could avert millions of fatalities and enhance the health and well-being of billions of people using polluting energy sources for cooking, heating, and lighting systems [39]. Indoor Air Pollution from solid fuel combustion is a leading cause of sickness and mortality across the world, and the most vulnerable section among those are the women and older, living in rural areas for a prolonged time in developing countries like India, Pakistan, Myanmar, and other South-East Asian countries as per WHO (2016) [40].

This study looked into the association between Indoor Air Pollution and the cognitive function of rural Indian older women, which is a little-discussed in India and found that exposure to Indoor Air Pollution had a significant deleterious effect on elderly women's cognitive abilities and functioning, this result was consistent with a related previous study that focused on middle-aged and older adults [10]. The elderly women's cognitive abilities and functioning were negatively correlated with their exposure to Indoor Air Pollution. This finding was also similar to other recent studies conducted around the world [41]. We found that compared to middle aged women, the connection was stronger in older women aged 60 or above, which may contribute to their lifelong exposure and dependency on unclean sources of energy daily in the rural settings of India, which was consistent with older adult’s prior studies [15]. Irrespective of the controlling effects of individual and household level factors separately or taken together, cognitive functioning was negatively affected by exposure to indoor air pollution. When individual factors were controlled for, educational status was positively associated with cognitive performance, which is also consistent with earlier research [22]. In addition, household-level socioeconomic status was negatively associated with cognition, especially among poorer households compared to non-poor households, this result is also consistent with findings from past research [10, 15]. As per the NSS survey (68th Round), over two-thirds of rural households depend on unclean fossil fuels like firewood for daily cooking as their first source of energy and other household activities [42]. Rural women spend their significant share of time in the kitchen for food and meal management and preparation [43] and are exposed to IAP on regular basis. When exposed to indoor air pollution, older women from rural areas who reported poorer self-rated health were more likely to have poorer cognitive functioning; this funding was consistent with prior research [10].

This study investigated the link between Indoor Air Pollution and the cognitive functioning health of rural Indian older women and found that exposure to indoor air pollution had a significant deleterious effect on elderly women's cognitive functioning health, indicating the negative correlation and confirming the hypothesis- poor cognitive functioning health among the women reported exposure to Indoor Air Pollution. Result from multi-level regression also showed that irrespective of the controlling effects of individual and household level factors separately or taken together, cognitive functioning was negatively associated with to indoor air pollution. This finding was also similar to other recent study conducted around the world [41].

However, on the enquire of individual factors, educational status was positively associated with cognitive performance, which is also consistent with earlier research [22]. In addition, household-level socioeconomic status was fond to be negatively associated with cognition, especially among poorer households compared to non-poor households. As per the NSS survey (68th Round), over two-thirds of rural households depend on unclean fossil fuels like firewood for daily cooking as their first source of energy and other household activities, therefore, it is obviously to expect poor cognitive functional health mong the poorer households [42]. It was also reported that rural women spend their significant share of time in the kitchen for food and meal management and preparation [43] and are exposed to IAP on regular basis. Rural older women who are reported to have better self-rated health are the ones who have relatively better cognitive functioning when exposed to indoor air pollution.

The above results highlighted some significant outcomes reported by various studies concerning indoor air pollution and cognitive functioning. Living in a poorer household significantly increases the chances of using a solid and unclean source of fuels in everyday life, and are thus exposed to many adverse outcomes. It is evident from the results that most rural households use unclean fuels and traditional stoves, don’t have a separate kitchen and have no ventilation system, which is prevalent in indoor air pollution. Thus, older adult women from disadvantaged backgrounds are more likely to suffer from cognitive dysfunction. Previous research also corroborates that individuals who live in poorer households and disadvantaged communities in India are the most vulnerable and exposed to ill health and mortality [10, 15, 44]. It is also evident from the earlier studies that due to culturally women are responsible for food preparation, they are more likely to be exposed to high levels of indoor smoke (such as exposure to carcinogens from domestic air pollution generated by cooking with kerosene or solid fuels like wood, charcoal, or coal) [39]. In the recent past, the Government of India launched the "Pradhan Mantri Ujjwala Yojana (PMUY)” to provide free LPG connections to poorer households across the country. However, studies found that loss of income due to the pandemic and other factors have resulted in lowered LPG subscriptions or stopped [45], with rural women resorting to traditional unclean energy sources for cooking daily meals.

### Strengths and limitations

The study's key strength is that it employed the latest national representative large-scale world most extensive elderly data from 2017–18 to answer the research issue. This study examined the association between indoor air pollution and middle-aged and older women in rural India, which the other studies have largely ignored. Hence, the findings of this study not only reveal current cognitive ability among rural middle-aged and older women but also throw some light on the association between cognitive functioning and their lifelong exposure to indoor air pollution.

Despite its many advantages, this study also suffered some unintended flaws. We were unable to address the cause-effect relationship of this association due to the cross-sectional nature of the dataset. In addition, because indoor air pollution was evaluated indirectly in the LASI survey, it is difficult to discern the degree of indoor air pollution due to data limitations. Furthermore, air pollution exposure tends to converge. We cannot be certain that the study results were only limited to indoor air pollution because there was no assessment of exterior air pollution or other chemicals to which women might have been exposed. Poor levels of health literacy among rural older women may also lead to misreporting of their cognitive health status and quality of life which can also affect the results.

### Implications for policy and further research scope

Since around 70 percent of the Indians reside in rural areas and 40 percent of the Indian households still dependent on unclean biomass fuel for domestic cooking purposes [30], India's policy actions are crucial for tackling indoor air pollution. Rural Indian has limited access to clean fuel; and due to economic burden, they are forced to use biomass; hence, indoor air pollution becomes a severe public health concern for the country. However, Indian government has taken various attempts (such as National Programme on Improved Chulha (NPIC) [46] and PMUY [45]) to ameliorate it. Studies must be conducted for adequate assessment and reconsideration of the price of LPG and subsidies for the poor, as the pandemic severely affected and decimated the income and livelihood of the rural people. Policymakers should also consider additional aspects such as raising knowledge about the adverse effects of utilising unclean biomass energy sources, indoor air pollution and the importance of having a proper ventilation system in the kitchen among rural women. Alternatives such as biomass-fuelled improved *chullha* with chimneys /cookstoves can be advocated in unavoidable situations, which may lead to lower indoor air pollution as evidenced by an earlier study [46, 47]. Furthermore, studies on the relationship between indoor air pollution and rural women's health, mainly cognitive function, are lacking; consequently, research in the near future should examine analysing this association using follow-up data and data from other middle-low-income countries.

## Conclusion

Despite numerous drawbacks, the present study provides persuasive evidence that indoor air pollution from solid and unclean cooking fuels may affect participants' cognitive ability. Given the social and economic costs associated with cognitive impairment in older persons, this study has significant public health implications. The present study suggests the awareness intervention in cognitive functioning health on the line of enhancing mental well-being and quality of life for the household members, especially for those spending prolonged time within household. It may help people be aware and avoid adverse outcomes from using solid fuels, not having ventilation, and smoke within the household from different sources. Indoor air pollution exposure can be reduced at a certain level by strengthening the ventilation system in the home and providing clean cooking fuel and improved stoves with chimneys for domestic usage. Therefore, the present study demands a program that may increase the availability and the accessibility of clean fuel and technologies that may also help country to go a step towards achieving SDG 7.

## Supplementary Information

**Additional file 1: Supplementary Table 1.** Multicollinearity check results. **Supplementary Table 2.** VIF value.

## Data Availability

The study is based on secondary data source, is freely available in the public domain through https://www.iipsindia.ac.in/lasi

## Abbreviations

IAP: Indoor Air Pollution
DALYs: Disability-Adjusted Life-Years
NSS: National Sample Survey
LASI: Longitudinal Ageing Study in India (LASI)
TB: Tuberculosis
LPG: Liquefied Petroleum Gas
