# Supplementary material for: Exposure to indoor air pollution and the cognitive functioning of elderly rural women: a cross-sectional study using LASI data, India
Source: BMC Public Health. 2022 Dec 5;22:2272. doi: 10.1186/s12889-022-14749-7 (PMC9724350; doi:10.1186/s12889-022-14749-7)
Supplement: Supplementary file 1 — Additional file 1: Supplementary Table 1. Multicollinearity check results. Supplementary Table 2. VIF value. [file 12889_2022_14749_MOESM1_ESM.docx]

Appendix

Supplementary Table -1: Multicollinearity check results

|  | Age group | Marital status | Years of schooling | Ever smoked or smokeless tobacco | Ever consumed alcohol | Living arrangement | SRH | Sleeping problem | MPCE | Religion | Region | Exposed to IAP |
| --- | --- | --- | --- | --- | --- | --- | --- | --- | --- | --- | --- | --- |
| Age group | -0.2854 | 1 |  |  |  |  |  |  |  |  |  |  |
| Marital status | -0.2222 | 0.332 | 1 |  |  |  |  |  |  |  |  |  |
| Years of schooling | 0.4832 | -0.1961 | -0.132 | 1 |  |  |  |  |  |  |  |  |
| Ever smoked or smokeless tobacco | 0.0975 | -0.0735 | -0.0945 | 0.0929 | 1 |  |  |  |  |  |  |  |
| Ever consumed alcohol | 0.0993 | -0.0008 | -0.0458 | 0.066 | 0.1513 | 1 |  |  |  |  |  |  |
| Living arrangement | 0.0588 | -0.1243 | -0.3076 | 0.0474 | 0.0233 | 0.0012 | 1 |  |  |  |  |  |
| SRH | -0.1201 | 0.1624 | 0.1039 | -0.0176 | -0.0569 | 0.0296 | -0.0381 | 1 |  |  |  |  |
| Sleeping problem | 0.0591 | -0.0449 | -0.0406 | 0.0241 | 0.0004 | -0.01 | 0.0264 | -0.124 | 1 |  |  |  |
| MPCE | 0.1469 | -0.038 | -0.0451 | 0.1821 | 0.0465 | 0.071 | -0.057 | 0.0254 | 0.0074 | 1 |  |  |
| Religion | 0.0107 | 0.0129 | 0.0067 | 0.0626 | 0.0167 | -0.0278 | 0.0103 | -0.017 | 0.0242 | 0.0765 | 1 |  |
| Region | 0.0524 | -0.0029 | 0.0583 | 0.1112 | 0.0335 | -0.0453 | -0.087 | -0.0245 | 0.0162 | 0.0446 | -0.0314 | 1 |
| Exposed to IAP | 0.1008 | -0.0288 | -0.0061 | 0.1148 | 0.001 | 0.0171 | 0.0211 | -0.0272 | 0.0135 | 0.1003 | -0.0284 | 0.1046 |

Supplementary Table-2: VIF value

| Variable | VIF |
| --- | --- |
|  |  |
| Age group | 1.25 |
| Marital status | 1.18 |
| Years of schooling | 1.12 |
| Ever smoked or smokeless tobacco | 1.11 |
| Ever consumed alcohol | 1.06 |
| living arrangement | 1.05 |
| SRH | 1.04 |
| Sleeping problem | 1.04 |
| MPCE | 1.04 |
| Religion | 1.03 |
| Region | 1.02 |
| Exposed to IAP | 1.02 |
|  |  |
| Mean VIF | 1.08 |
